# Detection of hepatitis B virus pre-S mutants in plasma by a next-generation sequencing-based platform determines their patterns in liver tissues

**DOI:** 10.1371/journal.pone.0234773

**Published:** 2020-06-19

**Authors:** Chiao-Fang Teng, Hung-Wen Tsai, Tsai-Chung Li, Ting Wang, John Wang, Woei-Cherng Shyu, Han-Chieh Wu, Ih-Jen Su, Long-Bin Jeng

**Affiliations:** 1 Graduate Institute of Biomedical Sciences, China Medical University, Taichung, Taiwan; 2 Organ Transplantation Center, China Medical University Hospital, Taichung, Taiwan; 3 Research Center for Cancer Biology, China Medical University, Taichung, Taiwan; 4 Department of Pathology, National Cheng Kung University Hospital, Tainan, Taiwan; 5 Department of Public Health, College of Public Health, China Medical University, Taichung, Taiwan; 6 Department of Healthcare Administration, College of Medical and Health Science, Asia University, Taichung, Taiwan; 7 Department of Pathology, China Medical University Hospital, Taichung, Taiwan; 8 Department of Occupational Therapy, Asia University, Taichung, Taiwan; 9 Department of Neurology, China Medical University Hospital, Taichung, Taiwan; 10 Translational Medicine Research Center, China Medical University Hospital, Taichung, Taiwan; 11 National Institute of Infectious Diseases and Vaccinology, National Health Research Institutes, Zhunan, Taiwan; 12 Department of Biotechnology, Southern Taiwan University of Science and Technology, Tainan, Taiwan; CEA, FRANCE

## Abstract

Hepatocellular carcinoma (HCC) is among the leading causes of cancer-related death worldwide. Patients with hepatitis B virus (HBV) pre-S mutants in liver tissues or blood have been regarded as a high-risk population for HCC development and recurrence. Detection of pre-S mutants in clinical specimens is thus important for early diagnosis and prognosis of HCC to improve patient survival. Recently, we have developed a next-generation sequencing (NGS)-based platform that can quantitatively detect pre-S mutants in patient plasma with superior sensitivity and accuracy. In this study, we compared the pre-S genotyping results from plasma by the NGS-based analysis with those from liver tissues by the immunohistochemistry (IHC)-based analysis in 30 HBV-related HCC patients. We demonstrated that the detection rate of pre-S mutants was significantly higher by NGS- than by IHC-based analysis. There was a moderate to good agreement between both analyses in detection of pre-S mutants. Compared with the IHC, the NGS-based detection of pre-S mutants in patient plasma could determine the patterns of pre-S mutants in liver tissues more efficiently in a noninvasive manner. Our data suggest that the NGS-based platform may represent a promising approach for detection of pre-S mutants as biomarkers of HBV-related HCC in clinical practice.

## Introduction

Hepatocellular carcinoma (HCC) is one of the most common and lethal human cancers worldwide, causing approximately 700,000 deaths per year [1–3]. Although liver transplantation and surgical resection are widely regarded as potentially curative treatments for HCC patients, the former is limited by the scarcity of donor livers and the latter is not suitable for every patient [4, 5]. Moreover, recurrence after curative resection of HCC is a frequent event, leading to poor patient survival [6, 7]. Currently available chemotherapeutic or molecular targeted drugs provide only limited survival benefit for HCC patients [8, 9]. Therefore, a reliable biomarker and its detection method are urgently needed for early diagnosis and prognosis of HCC to improve patient survival.

Chronic hepatitis B virus (HBV) infection is one of the major risk factors for HCC development, accounting for over 50% of total cases worldwide [10–12]. Our previous studies have identified that two different types of ground glass hepatocytes (GGH, designated types I and II) in liver tissues contain pre-S mutants harboring deletions in the pre-S1 (pre-S1 mutant) and pre-S2 (pre-S2 mutant) gene segments of HBV large surface antigen, respectively [13, 14]. As important HBV oncoproteins, pre-S mutants can induce multiple oncogenic signaling pathways, contributing to hepatocyte proliferation, genomic instability, and eventually HCC formation in vitro and in vivo [15–18]. Moreover, patients carrying pre-S mutants in liver tissues or blood have a 5-fold higher risk to develop HCC [19, 20], and represent a high-risk population for HCC recurrence after curative surgical resection even under antiviral treatment [21–23]. As a result, pre-S mutants have emerged as powerful biomarkers for HBV-related HCC.

Two methods have been commonly used to detect the presence of pre-S mutants in chronic HBV carriers and HBV-related HCC patients, one based on immunohistochemistry (IHC) staining of HBV surface antigen (HBsAg) for GGH visualization in liver tissues [14, 21, 22], and another TA cloning following polymerase chain reaction (PCR) amplification of pre-S gene for DNA sequencing in serum/plasma samples [13, 19, 20]. Unlike these two methods providing only qualitative and semi-quantitative results, we have recently developed a next-generation sequencing (NGS)-based platform for quantitative detection of pre-S mutants in patient plasma [24]. In contrast to the TA cloning-based method, the NGS-based platform can detect the presence of pre-S mutants (not only types but also levels) in patient plasma with higher sensitivity and accuracy [24]. However, it remains unclear whether the pre-S genotyping results by the NGS-based platform from the plasma of patients may correspond with those by the IHC-based method from the liver tissues.

**Fig 1:**
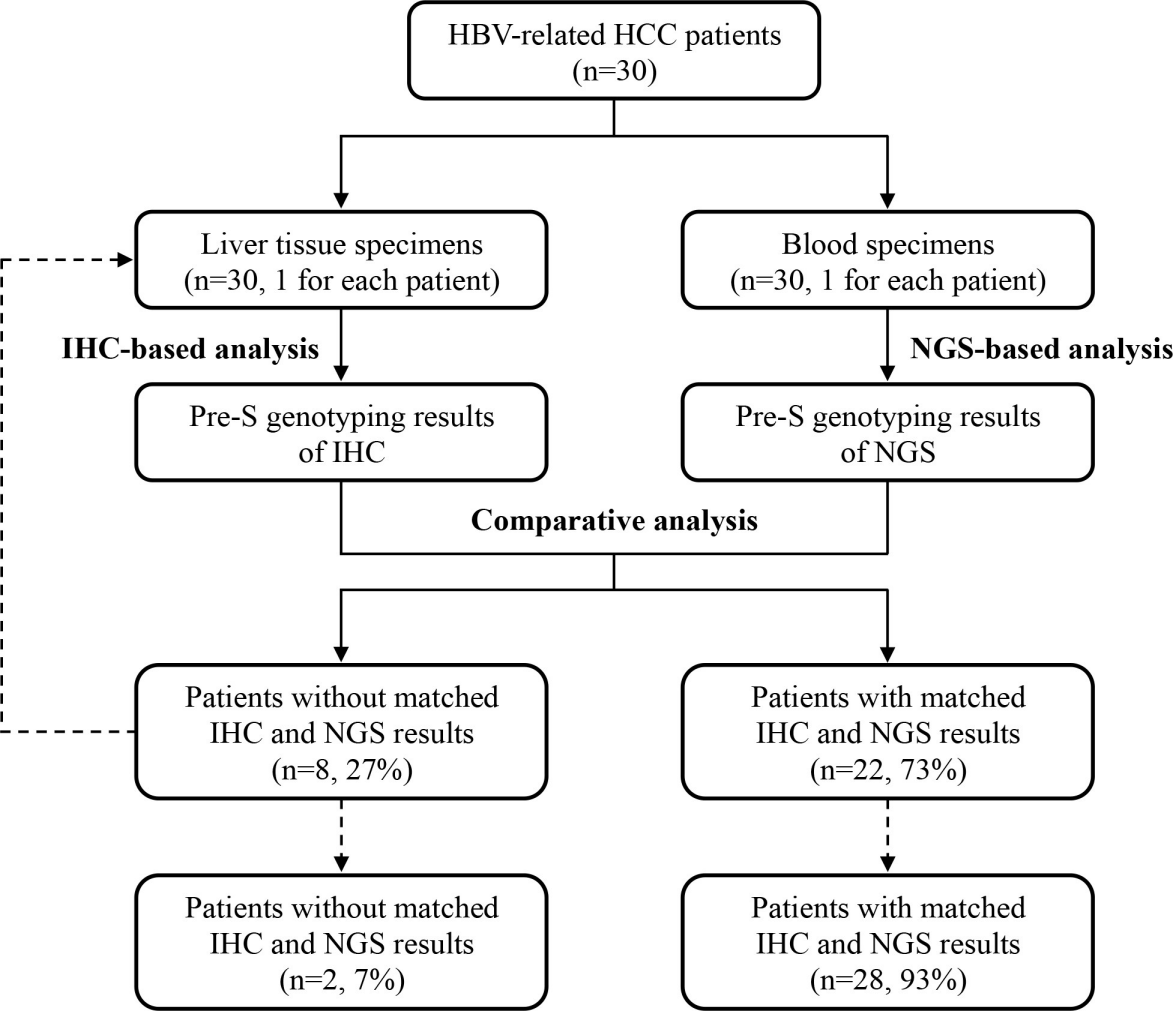
Flowchart for the comparison of pre-S genotyping results between liver tissues and plasma in this study. Liver tissue and plasma specimens collected from 30 HBV-related HCC patients (1 for each patient) were analyzed by IHC and NGS for detection of pre-S mutants, respectively. Comparative analysis of the pre-S genotyping results revealed that 22 out of 30 (73%) patients had matched IHC and NGS results but the other 8 (27%) did not. Further analysis of another 1 liver tissue specimen obtained from each of the 8 patients by IHC increased the number of the patients with matched results up to 28 (93%) (as indicated by dashed lines). The Abbreviations: HBV, hepatitis B virus; HCC, hepatocellular carcinoma; IHC, immunohistochemistry; NGS, next-generation sequencing.

To clarify this issue, in this study, we collected liver tissue and plasma specimens from 30 HBV-related HCC patients for detection of pre-S mutants by IHC- and NGS-based analyses, respectively (Fig 1). Furthermore, the pre-S genotyping results of IHC and NGS were comparatively analyzed to determine the percentage of patients with or without consistent patterns of pre-S mutants between liver tissues and plasma.

## Materials and methods

### Human specimens

The liver tissue and plasma specimens obtained during and before surgery, respectively, were collected retrospectively from 30 HBV-related HCC patients who underwent surgical treatment at China Medical University Hospital (Taichung, Taiwan), from Jan 2006 to Jul 2017, under the approval of the China Medical University & Hospital Research Ethics Committee (protocol No. CMUH107-REC1-080). The clinicopathological records of the patients were also collected.

### IHC-based pre-S genotyping analysis

The IHC-based detection of pre-S mutants was performed as previously described [21]. Briefly, liver tissue sections were incubated with the primary antibody anti-HBsAg (MA5-13059; Thermo Fisher Scientific, Rockford, IL, USA), followed by a horseradish peroxidase-conjugated secondary antibody. Subsequently, the sections were visualized with the aminoethyl carbazole substrate kit (Zymed Laboratories, San Francisco, CA, USA) and counterstained with hematoxylin. Finally, the types and scores of GGH were assessed by 2 pathologists (HWT and IJS). The percentage of each type of GGH was scored from 0 to 4 corresponding to 0%, <5%, 5% to 9%, 10% to 29%, and ≥30% of hepatocytes in liver tissues, respectively.

### NGS-based pre-S genotyping analysis

The NGS-based detection of pre-S mutants was performed as previously described [24]. Briefly, DNA isolated from plasma specimens was used as template for PCR amplification of the pre-S gene. Next, the pre-S gene PCR products were subjected to NGS sequencing analysis (Illumina, San Diego, CA, USA). Finally, the deletion types, regions, and levels of pre-S mutants were determined by using our customized scripts. The level of each type of pre-S mutant was defined as the number of pre-S gene DNA with deletion divided by the total number of pre-S gene DNA and then multiplied by 100.

### Statistical analysis

The difference between the pre-S genotyping results by IHC- and NGS-based analyses was compared by the McNemar’s paired proportion test, where a P value<0.05 was considered to indicate statistical significance. The agreement between both analyses in pre-S genotyping was evaluated by calculating the simple kappa coefficient with 95% CI, where κ = 1 denotes perfect agreement; κ>0.80, excellent; κ>0.60, good; κ>0.40, moderate; κ>0.20, fair; and κ>0 indicates poor agreement [25].

## Results

### Patient profile and clinicopathological data

The clinicopathological characteristics of the 30 HBV-related HCC patients enrolled in this study are summarized in Table 1. Among all patients, 26 (87%) were men, 4 (13%) were women, and the median age was 54 years (range, 28 to 78). 27 patients (90%) were HBsAg positive and 26 patients (87%) were HBV e antigen (HBeAg) negative. 24 patients (80%) had genotype B and 6 patients (20%) had genotype C HBV infection. HBV DNA was detected in 20 (67%) patients at a median of 6.8×10^4^ copies/mL (range, 30.1 to 1.5×10^8^). Tumor size was recorded in 29 (97%) patients at a median of 4.0 cm (range, 1.5 to 35.0).

**Table 1:** Clinicopathological characteristics of the 30 HBV-related HCC patients enrolled in this study.

Characteristics^a^	No. of Patients	Median (Range)
Age (years)	30	54 (28−78)
Gender (men/women)	26/4	
Smoking (yes/no)	10/20	
Alcohol (yes/no)	7/23	
HBsAg (positive/negative/NA)	27/0/3	
HBeAg (positive/negative)	4/26	
HBV genotype (B/C)	24/6	
HBV DNA (copies/mL) (20−1.7×10^8^/<20/NA)^b^	20/2/8	6.8×10^4^ (30.1−1.5×10^8^)
Albumin (g/dL)	30	3.7 (2.4−4.9)
AST (U/L)	30	53.5 (14.0−290.0)
ALT (U/L)	29	56 (13−292)
AFP (ng/mL) (≤54000/>54000)^c^	26/4	35.7 (2.4−4550.0)
Tumor size (cm)	29	4.0 (1.5−35.0)
Tumor encapsulation (yes/no)	22/7	
Lymph node involvement (yes/no)	4/26	
Portal vein thrombosis (yes/no)	0/30	
Satellite nodule (yes/no)	6/24	
Vascular invasion (yes/no)	14/16	
Distant metastasis (yes/no)	3/27	
Steatosis grade (0/1/2/3)	18/5/0/0	
Metavir inflammation score (0/1/2/3)	5/19/1/0	
Ishak fibrosis score (0/1/2/3/4/5/6)	1/5/5/8/5/1/4	
Child-Pugh cirrhosis score (A/B/C)	21/6/2	
CLIP score (0/1/2/3/4/5/6)	10/11/5/2/1/0/0	
BCLC stage (A/B/C/D)	18/6/4/2	
AJCC TNM stage (I/II/IIIA/IIIB/IIIC/IVA/IVB)	11/12/3/0/3/0/1	

^a^Only patients with available data were analyzed.

^b^HBV DNA was measured with a range of 20 to 1.7×10^8^ copies/mL.

^c^AFP was measured with the highest detection limit of 54000 ng/mL.

Abbreviations: HBV, hepatitis B virus; HCC, hepatocellular carcinoma; HBsAg; hepatitis B surface antigen; NA, not available; HBeAg, hepatitis B e antigen; AST, aspartate aminotransferase; ALT, alanine aminotransferase; AFP, alpha-fetoprotein; CLIP, Cancer of the Liver Italian Program; BCLC, Barcelona Clinic Liver Cancer; AJCC, American Joint Committee on Cancer; TNM, tumor-node-metastasis.

### IHC-based pre-S genotyping in liver tissues

By IHC-based pre-S genotyping analysis, we first detected pre-S mutants in liver tissues of the 30 HBV-related HCC patients. As shown in Table 2 and S1 Table, either or both of types I and II GGH were detected in 19 out of 30 (63%) patients, among whom 4 (13%) patients had only type I GGH (score 1–4), 6 (20%) patients had only type II GGH (score 1–4), and 9 (30%) patients had both type I GGH (score 1–4) and type II GGH (score 1–4). Additionally, 13 (43%) patients had type I GGH (score 1–4) without/with type II GGH, and 15 (50%) patients had type II GGH (score 1–4) without/with type I GGH.

**Table 2:** Summary of the pre-S genotyping results by IHC-based analysis in 30 HBV-related HCC patients.

Summary of The Pre-S Genotyping Results^a^	IHC-Based Analysis
Total patients (n) (%)	30 (100)
Patients without type I and type II GGH (n) (%)	11 (37)
Patients with type I and/or type II GGH (n) (%)	19 (63)
Patients with type I GGH (score 1–4) and type II GGH (score 0) (n) (%)	4 (13)
Patients with type I GGH (score 0) and type II GGH (score 1–4) (n) (%)	6 (20)
Patients with type I GGH (score 1–4) and type II GGH (score 1–4) (n) (%)	9 (30)
Patients with type I GGH (score 1–4) and type II GGH (score 0–4) (n) (%)	13 (43)
Patients with type II GGH (score 1–4) and type I GGH (score 0–4) (n) (%)	15 (50)

^a^The percentage of each type of GGH was scored from 0 to 4 corresponding to 0%, <5%, 5% to 9%, 10% to 29%, and ≥30% of hepatocytes in liver tissues, respectively.

Abbreviations: IHC, immunohistochemistry; GGH, ground glass hepatocytes; n, number; del, deletion.

### NGS-based pre-S genotyping in plasma

By NGS-based pre-S genotyping analysis, we next detected pre-S mutants in plasma of the 30 HBV-related HCC patients. As shown in Table 3 and S1 Table, 25 out of 30 (83%) patients had pre-S deletion, among whom 4 (13%) patients had only pre-S1 deletion, 7 (23%) patients had only pre-S2 deletion, 3 (10%) patients had both pre-S1 and pre-S2 deletion, 1 (3%) patient had both pre-S2 and pre-S1+pre-S2 deletion, and 10 (34%) patients had all three types of pre-S deletion. Moreover, 18 (60%) and 21 (70%) patients had deletion spanning the pre-S1 and pre-S2 gene segments, respectively.

**Table 3:** Summary of the pre-S genotyping results by NGS-based analysis in 30 HBV-related HCC patients.

Summary of The Pre-S Genotyping Results	NGS-Based Analysis
Total patients (n) (%)	30 (100)
Patients without pre-S del (n) (%)	5 (17)
Patients with pre-S del (n) (%)	25 (83)
Patients with only pre-S1 del (n) (%)	4 (13)
Patients with only pre-S2 del (n) (%)	7 (23)
Patients with only pre-S1+pre-S2 del (n) (%)	0 (0)
Patients with both pre-S1 and pre-S2 del (n) (%)	3 (10)
Patients with both pre-S1 and pre-S1+pre-S2 del (n) (%)	1 (3)
Patients with both pre-S2 and pre-S1+pre-S2 del (n) (%)	0 (0)
Patients with all three types of pre-S del (n) (%)	10 (34)
Patients with deletion spanning pre-S1 gene segment (n) (%)	18 (60)
Patients with deletion spanning pre-S2 gene segment (n) (%)	21 (70)

Abbreviations: NGS, next-generation sequencing; n, number; del, deletion.

### Detection of pre-S mutants by NGS in plasma determined their patterns in liver tissues with higher sensitivity and accuracy than by IHC

Comparative analysis of the pre-S genotyping results of IHC and NGS showed that 22 out of 30 (73%) patients had consistent results although the other 8 (27%) did not (S1 Table). In total 30 patients, pre-S mutants were detected in 19 (63%) and 25 (83%) patients by IHC and NGS, respectively (Tables 2 and 3). Six patients were negative for pre-S mutants by IHC-based analysis but conversely positive by NGS. The detection of pre-S mutants was significantly more sensitive by NGS- than by IHC-based analysis (McNemar’s paired proportion test, P value = 0.0143) (Table 4). The simple kappa value for the agreement between both analyses in detection of pre-S mutants was moderate at 0.51 (95% confidence interval (CI), 0.20 to 0.81) (Table 4). Similar results were observed when comparing both analyses in identifying patients with pre-S1 mutant (McNemar’s paired proportion test, P value = 0.0253; simple kappa coefficient, kappa value (κ) = 0.67 (95% CI, 0.42 to 0.92)) or pre-S2 mutant (McNemar’s paired proportion test, P value = 0.0143; simple kappa coefficient, κ = 0.60 (95% CI, 0.33 to 0.86)) (Table 4).

**Table 4:** Comparison of the pre-S genotyping results by IHC- and NGS-based analyses in 30 HBV-related HCC patients.

Patient groups by pre-S genotyping	McNemar’s paired proportion test				Simple Kappa Coefficient	
		NGS (−)	NGS (+)	P value	κ value	95% CI
Patients without (−)/with (+) any type of GGH/pre-S del (n) (%)	IHC (−)	5 (17)^a^	6 (20)^b^	0.0143	0.51	0.20–0.81
	IHC (+)	0 (0)^c^	19 (63)^d^			
Patients without (−)/with (+) type I GGH (score 1–4) and type II GGH (score 0–4)/deletion spanning pre-S1 gene segment (n) (%)	IHC (−)	12 (40)	5 (17)	0.0253	0.67	0.42–0.92
	IHC (+)	0 (0)	13 (43)			
Patients without (−)/with (+) type II GGH (score 1–4) and type I GGH (score 0–4)/deletion spanning pre-S2 gene segment (n) (%)	IHC (−)	9 (30)	6 (20)	0.0143	0.60	0.33–0.86
	IHC (+)	0 (0)	15 (50)			

^a^The number of patients without the indicated GGH and pre-S deletion by both IHC- and NGS-based analyses.

^b^The number of patients with the indicated pre-S deletion by NGS- but not IHC-based analysis.

^c^The number of patients with the indicated pre-S deletion by IHC- but not NGS-based analysis.

^d^The number of patients with the indicated pre-S deletion by both IHC- and NGS-based analyses.

Abbreviations: NGS, next-generation sequencing; GGH, ground glass hepatocytes; IHC, immunohistochemistry; del, deletion; n, number.

Considering that the IHC results were originally obtained from only 1 liver tissue specimen (a part of liver tissues) from each of the 30 HBV-related HCC patients, it could be reasonably speculated that the results might be insufficient to reflect the patterns of pre-S mutants in whole liver tissues. To further confirm the patterns of pre-S mutants in liver tissues, another 1 liver tissue specimen (a different part of liver tissues) was collected from each of the 8 patients with inconsistent IHC and NGS results for additional IHC-based analysis. As shown in Table 5, based on the IHC results from the original specimen, 6 out of the 8 patients did not have any type of GGH, and the other 2 had only type I GGH. Further analysis of another 1 specimen showed that 4 out of the 6 patients without any type of GGH as well as the 2 patients with only type I GGH became detectable for the types of GGH corresponding with the pre-S genotyping results of NGS (Table 5). Overall, the proportion of patients with matched IHC and NGS results could be increased from 22 of 30 (73%) patients by analyzing 1 liver tissue specimen from each patient up to 28 of 30 (93%) by including 2 liver tissue specimens in analysis.

**Table 5:** The pre-S genotyping results by additional IHC-based analysis in selected HBV-related HCC patients.

Patient No.	IHC Result (GGH Type (Score))^a^^,^^b^	NGS Result (Pre-S Deletion Type (%))^d^
4	1. type I GGH (0), type II GGH (0)	1. **pre-S1 del (86.404)**^**e**^
	2. **type I GGH (1), type II GGH (0)**^**c**^	2. **wild-type (12.695)**
		3. pre-S2 del (0.737)
		4. pre-S1+pre-S2 del (0.163)
7	1. type I GGH (3), type II GGH (0)	1. **pre-S1+pre-S2 del (46.237)**
	2. **type I GGH (3), type II GGH (3)**	2. **pre-S2 del (26.927)**
		3. **pre-S1 del (14.368)**
		4. **wild-type (12.467)**
9	1. type I GGH (0), type II GGH (0)	1. **pre-S2 del (56.155)**
	2. type I GGH (0), type II GGH (0)	2. **wild-type (42.610)**
		3. pre-S1+pre-S2 del (0.718)
		4. pre-S1 del (0.516)
13	1. type I GGH (0), type II GGH (0)	1. **wild-type (94.701)**
	2. **type I GGH (1), type II GGH (0)**	2. **pre-S1 del (5.086)**
		3. pre-S2 del (0.137)
		4. pre-S1+pre-S2 del (0.077)
14	1. type I GGH (1), type II GGH (0)	1. **wild-type (69.001)**
	2. **type I GGH (1), type II GGH (3)**	2. **pre-S1 del (20.530)**
		3. **pre-S2 del (9.463)**
		4. pre-S1+pre-S2 del (1.006)
18	1. type I GGH (0), type II GGH (0)	1. **pre-S2 del (41.477)**
	2. **type I GGH (1), type II GGH (4)**	2. **pre-S1+pre-S2 del (39.126)**
		3. **wild-type (12.348)**
		4. **pre-S1 del (7.048)**
20	1. type I GGH (0), type II GGH (0)	1. **wild-type (30.944)**
	2. **type I GGH (1), type II GGH (1)**	2. **pre-S2 del (30.409)**
		3. **pre-S1+pre-S2 del (29.105)**
		4. **pre-S1 del (9.542)**
26	1. type I GGH (0), type II GGH (0)	1. **wild-type (30.973)**
	2. type I GGH (0), type II GGH (0)	2. **pre-S1+pre-S2 del (27.774)**
		3. **pre-S1 del (27.161)**
		4. **pre-S2 del (14.091)**

^a^The percentage of each type of GGH was scored from 0 to 4 corresponding to 0%, <5%, 5% to 9%, 10% to 29%, and ≥30% of hepatocytes in liver tissues, respectively.

^b^The results of two liver tissue specimens (the original one and another one) from each patient were shown in descending order.

^c^The result of IHC in correspondence with that of NGS was shown in bold.

^d^The total percentage of pre-S gene DNA in each type of pre-S deletion was shown in descending order.

^e^The pre-S deletion type above the cut-off percentage (5.049) was shown in bold.

Abbreviations: IHC, immunohistochemistry; NGS, next-generation sequencing; GGH, ground glass hepatocytes; del, deletion.

## Discussion

Despite substantial progress in treatment, high morbidity and mortality of HCC in patients with chronic HBV infection are still a significant health problem [26–28]. Therefore, early diagnosis and prognosis of HCC by reliable biomarkers remain a key goal for improving patient survival. The presence of pre-S mutants in liver tissues or blood has emerged as a valuable biomarker for HBV-related HCC [19–23]. Recently, we have developed a NGS-based platform for quantitative detection of pre-S mutants in patient plasma with high sensitivity and accuracy using easy and relatively less invasive methods [24]. In this study, we further demonstrated that the NGS-based pre-S genotyping in plasma could also efficiently determine the patterns of pre-S mutants in liver tissues in HBV-related HCC patients.

The presence of pre-S mutants in chronic HBV carriers and HBV-related HCC patients has been commonly detected from the serum/plasma and liver tissue specimens by the TA cloning- and IHC-based analyses, respectively [13, 14, 19–22]. However, these two methods provide only qualitative and semi-quantitative results and remain to be optimized in practical operation. The TA cloning-based analysis is mainly dependent on cloning and sequencing of pre-S gene PCR bands that are clearly separated and visualized in agarose gel; the unseparated or invisible PCR bands may be omitted during the procedure, thus leading to lower detection sensitivity [24]. Also, the cloning procedure is somewhat time-consuming. In addition, the IHC-based analysis relies majorly on staining and visualization of GGH in sections of liver tissue specimens that are parts of whole liver tissues; the results from partial liver tissues may insufficiently represent those from whole liver tissues, thus resulting in false-negative results. In supportive of this notion, in this study we showed that the detection rate of GGH in total 2 liver tissue specimens was considerably higher than that in only 1 liver tissue specimen in the same patient. Furthermore, another limitation for the IHC-based analysis is the need of invasive liver biopsy that may have potential adverse effects on some patients [29]. The IHC-based analysis is somewhat time- and manpower-consuming, requiring skillful surgeons for the operation, technicians for tissue preparation process then IHC staining, and experienced pathologists for data interoperation.

To optimize the methodology for detection of pre-S mutants in patient specimens, we have recently developed a NGS-based platform that can quantitatively detect the presence of each type of pre-S mutant with higher sensitivity and accuracy than the TA cloning-based analysis in the plasma of HBV-related HCC patients [24]. In this study, we further compared the pre-S genotyping results of the NGS-based analysis in plasma with those of the IHC-based analysis in liver tissues (1 specimen for each patient) and showed a moderate to good agreement between both analyses in detection of pre-S mutants. Although the NGS exhibited significantly higher sensitivity than the IHC, the detection rate of pre-S mutants by IHC could be remarkably improved by including another 1 liver tissue specimen from each patient in analysis, leading to a higher level of consistency with the results of NGS-based analysis in plasma. Therefore, our data suggest that the NGS-based detection of pre-S mutants in plasma of patients can also determine the presence and pattern of pre-S mutants in liver tissues with better sensitivity and accuracy than the IHC-based analysis. Moreover, the detection procedure of the NGS-based analysis is somewhat more safe and efficient because there is no need for invasive liver biopsy to obtain more than one liver tissue specimens for IHC analysis; the NGS-based analysis only requires some plasma and technicians for a NGS run that should be able to get the results within 24 hours if the workflow is well established. Not only the NGS-based analysis saves time, but also providing a nonbiased overall assessment for pre-S mutant detection with better accuracy and safety.

## Conclusions

In this study we demonstrated that the NGS-based pre-S genotyping in plasma could serve as an alternative approach to determine the presence and pattern of pre-S mutants in liver tissues in HBV-related HCC patients. Considering the advantages of high sensitivity and noninvasive procedure in detection of pre-S mutants, the NGS-based platform may have great promise for future clinical application in patients with chronic HBV infection or HBV-related HCC.

## Supporting information

S1 Table List of the pre-S genotyping results by IHC- and NGS-based analyses in 30 HBV-related HCC patients. (DOCX)
