# Supplementary material for: Detection of hepatitis B virus pre-S mutants in plasma by a next-generation sequencing-based platform determines their patterns in liver tissues
Source: PLoS One. 2020 Jun 19;15(6):e0234773. doi: 10.1371/journal.pone.0234773 (PMC7304603; doi:10.1371/journal.pone.0234773)
Supplement: S1 Table — (DOCX) [file pone.0234773.s001.docx]

**S1 Table.** **List of the pre-S genotyping results by IHC- and NGS-based analyses in 30 HBV-related HCC patients**

| Patient No. | IHC Result (GGH Type (Score))^a^ | NGS Result (Pre-S Deletion Type (%))^b^ | NGS Result (Pre-S Deletion Region (%))^c^ |
| --- | --- | --- | --- |
| 1 | 1. type I GGH (3)  2. type II GGH (3) | 1. **pre-S1 del (76.228)^d^**  2. **wild-type (12.583)**  3. **pre-S2 del (10.622)**  4. pre-S1+pre-S2 del (0.567) | 1. pre-S1 del (nt 3110-3127) (24.869)  2. wild-type (12.583)  3. pre-S2 del (nt 1-57) (10.000)  4. pre-S1+pre-S2 del (nt 2855-2872, 1-57) (0.490) |
| 2 | 1. type I GGH (2)  2. type II GGH (0) | 1. **wild-type (71.590)**  2. **pre-S1 del (28.270)**  3. pre-S2 del (0.129)  4. pre-S1+pre-S2 del (0.011) | 1. wild-type (71.590)  2. pre-S1 del (nt 2854-3147) (23.961)  3. pre-S2 del (nt 3211-3216) (0.048)  4. pre-S1+pre-S2 del (nt 2954-3097, 1-54) (0.002) |
| 3 | 1. type I GGH (0)  2. type II GGH (4) | 1. **wild-type (58.461)**  2. **pre-S2 del (37.194)**  3. pre-S1 del (2.939)  4. pre-S1+pre-S2 del (1.407) | 1. wild-type (58.461)  2. pre-S2 del (nt 24-50) (33.801)  3. pre-S1 del (nt 2880-3146) (0.646)  4. pre-S1+pre-S2 del (nt 2880-3146, 24-50) (0.355) |
| 4*^e^ | 1. type I GGH (0)  2. type II GGH (0) | 1. **pre-S1 del (86.404)**  2. **wild-type (12.695)**  3. pre-S2 del (0.737)  4. pre-S1+pre-S2 del (0.163) | 1. pre-S1 del (nt 3110-3127) (26.821)  2. wild-type (12.695)  3. pre-S2 del (nt 1-12) (0.219)  4. pre-S1+pre-S2 del (nt 2855-2872, 1-54) (0.016) |
| 5 | 1. type I GGH (0)  2. type II GGH (0) | 1. **wild-type (99.000)**  2. pre-S1 del (0.927)  3. pre-S2 del (0.062)  4. pre-S1+pre-S2 del (0.012) | 1. wild-type (99.000)  2. pre-S1 del (nt 3106-3129) (0.294)  3. pre-S2 del (nt 1-54) (0.031)  4. pre-S1+pre-S2 del (nt 2855-2972, 1-54) (0.008) |
| 6 | 1. type I GGH (0)  2. type II GGH (1) | 1. **wild-type (59.263)**  2. **pre-S2 del (37.934)**  3. pre-S1 del (1.940)  4. pre-S1+pre-S2 del (0.862) | 1. wild-type (59.263)  2. pre-S2 del (nt 1-15) (36.325)  3. pre-S1 del (nt 3026-3205) (1.007)  4. pre-S1+pre-S2 del (nt 3106-3126, 1-15) (0.439) |

**S1 Table. List of the pre-S genotyping results by IHC- and NGS-based analyses in 30 HBV-related HCC patients (continued)**

| Patient No. | IHC Result (GGH Type (Score))^a^ | NGS Result (Pre-S Deletion Type (%))^b^ | NGS Result (Pre-S Deletion Region (%))^c^ |
| --- | --- | --- | --- |
| 7* | 1. type I GGH (3)  2. type II GGH (0) | 1. **pre-S1+pre-S2 del (46.237)**  2. **pre-S2 del (26.927)**  3. **pre-S1 del (14.368)**  4. **wild-type (12.467)** | 1. pre-S1+pre-S2 del (nt 2956-3126, 1-9) (24.002)  2. pre-S2 del (nt 1-54) (23.226)  3. pre-S1 del (nt 2944-3075) (5.140)  4. wild-type (12.467) |
| 8 | 1. type I GGH (0)  2. type II GGH (0) | 1. **wild-type (97.851)**  2. pre-S1 del (2.037)  3. pre-S2 del (0.108)  4. pre-S1+pre-S2 del (0.005) | 1. wild-type (97.851)  2. pre-S1 del (nt 3026-3205) (1.109)  3. pre-S2 del (nt 3211-3213) (0.049)  4. pre-S1+pre-S2 del (nt 2855-2872, 1-54) (0.003) |
| 9* | 1. type I GGH (0)  2. type II GGH (0) | 1. **pre-S2 del (56.155)**  2. **wild-type (42.610)**  3. pre-S1+pre-S2 del (0.718)  4. pre-S1 del (0.516) | 1. pre-S2 del (nt 49-54) (56.043)  2. wild-type (42.610)  3. pre-S1+pre-S2 del (nt 3105-3126, 49-53) (0.251)  4. pre-S1 del (nt 3105-3128) (0.103) |
| 10 | 1. type I GGH (1)  2. type II GGH (0) | 1. **pre-S1 del (76.151)**  2. **wild-type (19.034)**  3. pre-S2 del (4.624)  4. pre-S1+pre-S2 del (0.192) | 1. pre-S1 del (nt 3110-3127) (16.407)  2. wild-type (19.034)  3. pre-S2 del (nt 1-54) (4.081)  4. pre-S1+pre-S2 del (nt 2855-2872, 2897-2923, 1-54) (0.051) |
| 11 | 1. type I GGH (0)  2. type II GGH (0) | 1. **wild-type (98.964)**  2. pre-S1 del (0.943)  3. pre-S2 del (0.084)  4. pre-S1+pre-S2 del (0.009) | 1. wild-type (98.964)  2. pre-S1 del (nt 2854-2970) (0.125)  3. pre-S2 del (nt 1-9) (0.028)  4. pre-S1+pre-S2 del (nt 2954-3097, 1-13) (0.009) |
| 12 | 1. type I GGH (0)  2. type II GGH (0) | 1. **wild-type (97.781)**  2. pre-S1 del (2.099)  3. pre-S2 del (0.112)  4. pre-S1+pre-S2 del (0.008) | 1. wild-type (97.781)  2. pre-S1 del (nt 3026-3205) (1.099)  3. pre-S2 del (nt 3211-3216) (0.055)  4. pre-S1+pre-S2 del (nt 3026-3205, 43-138) (0.002) |

**S1 Table. List of the pre-S genotyping results by IHC- and NGS-based analyses in 30 HBV-related HCC patients (continued)**

| Patient No. | IHC Result (GGH Type (Score))^a^ | NGS Result (Pre-S Deletion Type (%))^b^ | NGS Result (Pre-S Deletion Region (%))^c^ |
| --- | --- | --- | --- |
| 13* | 1. type I GGH (0)  2. type II GGH (0) | 1. **wild-type (94.701)**  2. **pre-S1 del (5.086)**  3. pre-S2 del (0.137)  4. pre-S1+pre-S2 del (0.077) | 1. wild-type (94.701)  2. pre-S1 del (nt 3026-3205) (2.765)  3. pre-S2 del (nt 51-143) (0.030)  4. pre-S1+pre-S2 del (nt 2854-2997, 25-128) (0.014) |
| 14* | 1. type I GGH (1)  2. type II GGH (0) | 1. **wild-type (69.001)**  2. **pre-S1 del (20.530)**  3**. pre-S2 del (9.463)**  4. pre-S1+pre-S2 del (1.006) | 1. wild-type (69.001)  2. pre-S1 del (nt 3110-3127) (4.779)  3. pre-S2 del (nt 1-54) (8.226)  4. pre-S1+pre-S2 del (nt 2855-2872, 1-18, 26-59) (0.259) |
| 15 | 1. type I GGH (2)  2. type II GGH (3) | 1. **wild-type (50.938)**  2. **pre-S1 del (19.760)**  3. **pre-S1+pre-S2 del (15.021)**  4. **pre-S2 del (14.280)** | 1. wild-type (50.938)  2. pre-S1 del (nt 2854-2970) (5.470)  3. pre-S1+pre-S2 del (nt 2855-2872, 1-54) (12.421)  4. pre-S2 del (nt 1-54) (13.606) |
| 16 | 1. type I GGH (1)  2. type II GGH (1) | 1. **wild-type (75.230)**  2. **pre-S2 del (13.878)**  3. **pre-S1 del (10.730)**  4. pre-S1+pre-S2 del (0.162) | 1. wild-type (75.230)  2. pre-S1+pre-S2 del (nt 2860-2880, 2954-3097, 1-15) (11.572)  3. pre-S1 del (nt 2860-2880, 2954-3097) (9.550)  4. pre-S2 del (nt 1-15) (0.138) |
| 17 | 1. type I GGH (0)  2. type II GGH (0) | 1. **wild-type (98.069)**  2. pre-S1 del (1.263)  3. pre-S2 del (0.609)  4. pre-S1+pre-S2 del (0.059) | 1. wild-type (98.069)  2. pre-S1 del (nt 3103-3126) (0.253)  3. pre-S2 del (nt 1-15) (0.535)  4. pre-S1+pre-S2 del (nt 2854-2996, 44-144) (0.015) |
| 18* | 1. type I GGH (0)  2. type II GGH (0) | 1. **pre-S2 del (41.477)**  2. **pre-S1+pre-S2 del (39.126)**  3. **wild-type (12.348)**  4. **pre-S1 del (7.048)** | 1. pre-S2 del (nt 1-54) (24.620)  2. pre-S1+pre-S2 del (nt 2855-2872, 1-54) (26.451)  3. wild-type (12.348)  4. pre-S1 del (nt 2855-2872) (4.978) |

**S1 Table. List of the pre-S genotyping results by IHC- and NGS-based analyses in 30 HBV-related HCC patients (continued)**

| Patient No. | IHC Result (GGH Type (Score))^a^ | NGS Result (Pre-S Deletion Type (%))^b^ | NGS Result (Pre-S Deletion Region (%))^c^ |
| --- | --- | --- | --- |
| 19 | 1. type I GGH (3)  2. type II GGH (3) | 1. **pre-S1+pre-S2 del (45.703)**  2. **pre-S2 del (29.458)**  3. **wild-type (14.926)**  4. **pre-S1 del (9.913)** | 1. pre-S1+pre-S1 del (nt 2855-2872, 1-18, 26-59) (20.608)  2. pre-S2 del (nt 1-18, 26-59) (15.861)  3. wild-type (14.926)  4. pre-S1 del (nt 2855-2872) (6.398) |
| 20* | 1. type I GGH (0)  2. type II GGH (0) | 1. **wild-type (30.944)**  2. **pre-S2 del (30.409)**  3. **pre-S1+pre-S2 del (29.105)**  4. **pre-S1 del (9.542)** | 1. wild-type (30.944)  2. pre-S2 del (nt 1-54) (19.525)  3. pre-S1+pre-S2 del (nt 2855-2872, 1-54) (23.945)  4. pre-S1 del (nt 2855-2872) (8.283) |
| 21 | 1. type I GGH (0)  2. type II GGH (4) | 1. **pre-S2 del (94.816)**  2. **wild-type (5.049)**  3. pre-S1+pre-S2 del (0.070)  4. pre-S1 del (0.066) | 1. pre-S2 del (nt 1-54) (94.697)  2. wild-type (5.049)  3. pre-S1+pre-S2 del (nt 3152-3202, 1-54) (0.023)  4. pre-S1 del (nt 3108-3137) (0.011) |
| 22 | 1. type I GGH (0)  2. type II GGH (3) | 1. **wild-type (65.077)**  2. **pre-S2 del (28.468)**  3. pre-S1 del (4.516)  4. pre-S1+pre-S2 del (1.940) | 1. wild-type (65.077)  2. pre-S2 del (nt 1-54) (17.044)  3. pre-S1 del (nt 2855-2872) (1.096)  4. pre-S1+pre-S2 del (nt 2855-2872, 1-54) (1.471) |
| 23 | 1. type I GGH (0)  2. type II GGH (3) | 1. **wild-type (91.356)**  2. **pre-S2 del (8.015)**  3. pre-S1 del (0.379)  4. pre-S1+pre-S2 del (0.251) | 1. wild-type (91.356)  2. pre-S2 del (nt 1-54) (3.969)  3. pre-S1 del (nt 2854-3021) (0.170)  4. pre-S1+pre-S2 del (nt 2855-2872, 1-54) (0.037) |
| 24 | 1. type I GGH (2)  2. type II GGH (3) | 1. **pre-S1 del (40.086)**  2. **wild-type (34.919)**  3. **pre-S2 del (16.065)**  4. **pre-S1+pre-S2 del (8.930)** | 1. pre-S1 del (nt 2855-2965) (24.125)  2. wild-type (34.919)  3. pre-S2 del (nt 1-54) (15.565)  4. pre-S1+pre-S2 del (nt 3022-3126, 1-60) (2.911) |

**S1 Table. List of the pre-S genotyping results by IHC- and NGS-based analyses in 30 HBV-related HCC patients (continued)**

| Patient No. | IHC Result (GGH Type (Score))^a^ | NGS Result (Pre-S Deletion Type (%))^b^ | NGS Result (Pre-S Deletion Region (%))^c^ |
| --- | --- | --- | --- |
| 25 | 1. type I GGH (1)  2. type II GGH (3) | 1. **pre-S1 del (29.181)**  2. **wild-type (23.836)**  3. **pre-S2 del (23.645)**  4. **pre-S1+pre-S2 del (23.338)** | 1. pre-S1 del (nt 2856-2969) (17.015)  2. wild-type (23.836)  3. pre-S2 del (nt 1-54) (23.402)  4. pre-S1+pre-S2 del (nt 2855-2872, 1-54) (15.956) |
| 26* | 1. type I GGH (0)  2. type II GGH (0) | 1. **wild-type (30.973)**  2. **pre-S1+pre-S2 del (27.774)**  3. **pre-S1 del (27.161)**  4. **pre-S2 del (14.091)** | 1. wild-type (30.973)  2. pre-S1+pre-S2 del (nt 2855-2872, 1-54) (24.398)  3. pre-S1 del (nt 2854-2970) (14.813)  4. pre-S2 del (nt 1-54) (13.846) |
| 27 | 1. type I GGH (1)  2. type II GGH (2) | 1. **wild-type (46.914)**  2. **pre-S1 del (45.517)**  3. **pre-S2 del (6.834)**  4. pre-S1+pre-S2 del (0.735) | 1. wild-type (46.914)  2. pre-S1 del (nt 2854-2970) (37.666)  3. pre-S2 del (nt 1-54) (5.216)  4. pre-S1+pre-S2 del (nt 2854-2970, 1-54) (0.307) |
| 28 | 1. type I GGH (1)  2. type II GGH (3) | 1. **pre-S1 del (43.130)**  2. **wild-type (41.965)**  3. **pre-S2 del (9.508)**  4. **pre-S1+pre-S2 del (5.397)** | 1. pre-S1 del (nt 2854-2970) (35.888)  2. wild-type (41.965)  3. pre-S2 del (nt 1-54) (9.239)  4. pre-S1+pre-S2 del (nt 2855-2872, 1-54) (4.160) |
| 29 | 1. type I GGH (1)  2. type II GGH (2) | 1. **wild-type (36.868)**  2. **pre-S1 del (35.238)**  3. **pre-S2 del (21.130)**  4. **pre-S1+pre-S2 del (6.763)** | 1. wild-type (36.868)  2. pre-S1 del (nt 2854-2970) (29.499)  3. pre-S2 del (nt 1-54) (20.218)  4. pre-S1+pre-S2 del (nt 2855-2872, 1-54) (3.332) |
| 30 | 1. type I GGH (0)  2. type II GGH (4) | 1. **wild-type (58.984)**  2. **pre-S2 del (34.533)**  3. pre-S1+pre-S2 del (4.497)  4. pre-S1 del (1.986) | 1. wild-type (58.984)  2. pre-S2 del (nt 1-57) (20.926)  3. pre-S1+pre-S2 del (nt 3026-3205, 2-55) (0.590)  4. pre-S1 del (nt 2944-3120) (1.128) |

**S1 Table. List of the pre-S genotyping results by IHC- and NGS-based analyses in 30 HBV-related HCC patients (continued)**

^a^The percentage of each type of GGH was scored from 0 to 4 corresponding to 0%, <5%, 5% to 9%, 10% to 29%, and ≥30%, respectively.

^b^The total percentage of pre-S gene DNA in each type of pre-S deletion was shown in descending order.

^c^The pre-S gene DNA with the highest percentage in each type of pre-S deletion was shown.

^d^The pre-S deletion type above the cut-off percentage (5.049) was shown in bold.

^e^The patients without matched IHC and NGS results were highlighted by asterisk.

Abbreviations: IHC, immunohistochemistry; GGH, ground glass hepatocytes; NGS, next-generation sequencing; del, deletion; nt, nucleotide.
